# Supplementary material for: Distance from the Community to the City Center Is a Vital Determinant of Dietary Diversity Score for Rural Community-Dwelling Older Adults in Taiwan
Source: Nutrients. 2025 Apr 10;17(8):1318. doi: 10.3390/nu17081318 (PMC12030559; doi:10.3390/nu17081318)
Supplement: Supplementary file 1 [file nutrients-17-01318-s001.zip › nutrients-3549185-supplementary.pdf]

Supplement Table S1. Distribution of BMI.

BMI	N	%
Underweight (BMI<18.5)	20	3.5
Normal (18.5≤BMI<24)	241	42.5
Overweight (24≤BMI<27)	176	31.0
Obesity (27 ≤ BMI)	130	22.9
	567	100

Supplement Table S2. Distribution of Calorie intake(kcal/day).

Recommended calorie intake	N	%
≥ 75%	287	50.6
< 75%	251	44.3
Missing	29	5.1
	567	100
Protein intake	N	%
≥ 0.8g/kg	293	51.7
<0.8g/kg	245	43.2
Missing	29	5.1
	567	100

Insufficient calorie intake is defined as consuming less than 75% of the recommended daily intake, while insufficient protein intake is defined as consuming less than 0.8 g/kg of body weight.
